# The *Rickettsia* Endosymbiont of *Ixodes pacificus* Contains All the Genes of De Novo Folate Biosynthesis

**DOI:** 10.1371/journal.pone.0144552

**Published:** 2015-12-09

**Authors:** Daniel J. Hunter, Jessica L. Torkelson, James Bodnar, Bobak Mortazavi, Timothy Laurent, Jeff Deason, Khanhkeo Thephavongsa, Jianmin Zhong

**Affiliations:** 1 Department of Biological Sciences, Humboldt State University, Arcata, California, United States of America; 2 Center for Outcomes Research and Evaluation, Yale School of Medicine, New Haven, Connecticut, United States of America; University of Maryland, College Park, UNITED STATES

## Abstract

Ticks and other arthropods often are hosts to nutrient providing bacterial endosymbionts, which contribute to their host’s fitness by supplying nutrients such as vitamins and amino acids. It has been detected, in our lab, that *Ixodes pacificus* is host to *Rickettsia* species phylotype G021. This endosymbiont is predominantly present, and 100% maternally transmitted in *I*. *pacificus*. To study roles of phylotype G021 in *I*. *pacificus*, bioinformatic and molecular approaches were carried out. MUMmer genome alignments of whole genome sequence of *I*. *scapularis*, a close relative to *I*. *pacificus*, against completely sequenced genomes of *R*. *bellii* OSU85-389, *R*. *conorii*, *and R*. *felis*, identified 8,190 unique sequences that are homologous to *Rickettsia* sequences in the NCBI Trace Archive. MetaCyc metabolic reconstructions revealed that all folate gene orthologues (*folA*, *folC*, *folE*, *folKP*, *ptpS)* required for de novo folate biosynthesis are present in the genome of *Rickettsia buchneri* in *I*. *scapularis*. To examine the metabolic capability of phylotype G021 in *I*. *pacificus*, genes of the folate biosynthesis pathway of the bacterium were PCR amplified using degenerate primers. BLAST searches identified that nucleotide sequences of the *folA*, *folC*, *folE*, *folKP*, and *ptpS* genes possess 98.6%, 98.8%, 98.9%, 98.5% and 99.0% identity respectively to the corresponding genes of *Rickettsia buchneri*. Phylogenetic tree constructions show that the folate genes of phylotype G021 and homologous genes from various *Rickettsia* species are monophyletic. This study has shown that all folate genes exist in the genome of *Rickettsia* species phylotype G021 and that this bacterium has the genetic capability for de novo folate synthesis.

## Introduction

Symbiotic relationships with microorganisms are widespread across arthropods. It has been reported that all insects, and even a majority of arthropods, carry symbiotic microorganisms [1, 2]. Microbial symbionts vary greatly in the effects on their hosts, ranging from provision of essential nutrients [3–5] and symbiont-mediated protection from pathogens and parasites [6–8], to altering the host's reproductive system or sex determination systems [9, 10]. Recently, it was found that a symbiont-mediated stimulation of host immune system may contribute to host defense against pathogens and natural enemies [2, 11].

Most symbiotic relationships have a biochemical basis [12, 13]. For eukaryotic hosts with limited metabolic capabilities, some of the most discernable benefits of microbial symbionts in a mutualistic relationship involve the provision of essential amino acids [14, 15], vitamins and cofactors [16, 17], and recycling and storage of nitrogenous wastes from hosts [18, 19]. The exchange of these nutritional constituents appears to be a primary driving force for eukaryotic cell evolution [20].

Arthropods that feed primarily or entirely on blood are a breeding ground for forming nutritional interactions with microbial symbionts. For instance, vertebrate blood rarely contains sufficient quantities of essential B-vitamins, which are eight life-essential enzymes and cofactors as well as their derivatives, including biotin and folate [16]. Since B-vitamin synthetic pathways cannot be independently produced by animals [21], the diet of blood feeding arthropods is complemented with bacterially synthesized vitamins [16, 22, 23]. The western black-legged tick, *Ixodes pacificus*, is a common human biter in the Pacific Coast states of the U.S. [24]. *I*. *scapularis* is a closely related tick species of *I*. *pacificus* and is found on the east coast of North America. Both *I*. *pacificus* and *I*. *scapularis* are major vectors of tick-borne diseases, including Lyme borreliosis and anaplasmosis [25]. With limited feeding events during the life cycle, ticks’ sole diet is blood from vertebrates. However, the sources of B-vitamins for *I*. *pacificus* ticks are unknown. Due to their exclusive nutrient-poor vertebrate blood diets, ticks must obtain many vitamins through non-dietary means.

It has been detected, in our lab, that *I*. *pacificus* is host to a rickettsial endosymbiont, *Rickettsia* species phylotype G021 [26]. This endosymbiont is predominantly present, and 100% maternally transmitted [27, 28]. The function of the *Rickettsia* species phylotype G021 in *I*. *pacificus* is unknown, but the phylotype does not appear to have an effect on embryogenesis, oviposition and egg hatching of *I*. *pacificus* [29].

The acquisition of DNA sequences by the advent of rapid genome sequencing techniques has enabled substantial progress in identification of the biosynthetic capabilities of symbionts in arthropods [16, 23, 30, 31]. Since many bacterial symbionts are obligate intracellular organisms that reside exclusively inside host cells, analysis of many symbionts’ genomes is impeded by the inability to separate bacterial nucleotide sequences away from host’s genomes. One method of discovering genomic sequences of symbionts beyond the arthropods’ genomes that were originally sequenced is by MUMmer, a genome-wide alignment software [32, 33]. In this report, the genome-sequencing project of *I*. *scapularis* was used to reveal genomic sequences of the tick’s bacterial partner, the symbiotic *Rickettsia* species phylotype G021, by MUMmer alignment, and to demonstrate that the bacterium has all the genes for synthesizing folate in the tick host, *I*. *pacificus*.

## Materials and Methods

### Bioinformatics identification of folate biosynthetic genes

The *Ixodes scapularis* Genome Project is a partnership between the J. Craig Venter Institute, the National Institute Health, and the Broad Institute [34, 35]. In 2008, 570,640 contigs and 369,492 scaffolds of the *I*. *scapularis* Genome Project were deposited in the NCBI Trace Archive.

To test if there are *Rickettsia* sequences in *I*. *scapularis*’s Trace Archive database, MUMmer (mummer.sourceforge.net), which is a suffix-tree algorithm, was used to rapidly align the contigs and scaffolds of the *I*. *scapularis* genome against three reference *Rickettsia* genomes. Specifically, NUCMER from MUMmer was used to align nucleotide sequences between the *I*. *scapularis* genome and three completely sequenced *Rickettsia* genomes: *R*. *bellii* RML 369-C from *Rickettsia bellii* group *Rickettsia* [36], *R*. *conorii* subsp. israelensis [37], and *R*. *felis* str. LSU-Lb [38] from spotted fever group *Rickettsia* [39]. Nucleotide sequences of the *I*. *scapularis* genome with identities that were greater than 70% with either of the three reference *Rickettsia* genomes were retrieved by NUCMER.

Several java scripts were developed to manipulate data obtained from the MUMmer searches. The first program, SequenceSearch, takes call numbers from the MUMmer search and returns the matching DNA sequences as output files. A second program, called NucleotideReader, features the ability to read the input nucleotide file, communicate with the NCBI Basic Local Alignment Search Tool BLASTX website that aligns translated DNA with proteins from a non-redundant protein database, and identify the top protein match on the result output HTML of BLASTX and return that protein name. NucleotideReader works for large-batch searches (>5000) and is very useful when analyzing large data sets (S1 File and S2 File). Finally vitamin biosynthesis pathways were created manually from the genome of *Rickettsia* species by MetaCyc (http://metacyc.org/META/NEW-IMAGE?object=Vitamin-Biosynthesis), a manual curated database containing 2,260 metabolic pathways from 2,600 organisms. The names of the proteins in each vitamin biosynthesis pathway were matched against those in the BLASTX output files. The names and EC numbers of the matched proteins from MetaCyc were recorded.

### Tick collection and Identification

*I*. *pacificus* adult ticks were collected from rural areas of Humboldt County in Northern California (UTM coordinates: Northing 4530184; Easting 567363; Zone/Sector 51T) by flagging or sweeping grass blades and shrubs with a 1-m^2^ white cotton cloth. Ticks were classified to the genus and species level based on morphological characteristics [40].

### DNA Extraction Method

Ticks were washed in 95% ethanol three times and DNA from the ticks was extracted using the ammonium hydroxide DNA extraction method [41]. Briefly, whole *I*. *pacificus* adult ticks were submerged in liquid nitrogen and homogenized to a fine powder. Cells were lysed by 0.7 M ammonium hydroxide and boiled at 100°C for 15 minutes, cooled on ice for 30 seconds and the remaining ammonium was allowed to evaporate from the open tube for 15 minutes. DNA concentrations were determined using a Nanodrop ND-1000 spectrophotometer (Thermo Fisher Scientific, Hampton, NH) and DNA was stored at -20°C.

### Polymerase Chain Reaction (PCR) primer design and PCR

To amplify whole open reading frames (ORFs) of the folate genes of *Rickettsia* species phylotype G021, conserved regions that are upstream and downstream of each folate gene among at least nine spotted fever group rickettsiae were identified by BLAST using folate gene sequences of *R*. *buchneri* in *I*. *scapularis* from the Trace Archive and aligned using alignment software, Codon Code Aligner 3.7.1.2 (http://www.codoncode.com/aligner/) and ClustalX version 2.0. The identified conserved regions were then used as templates for the primer design by Primer3 (www.simgene.com/Primer3) (Table 1).

**Table 1:** Oligonucleotide primers used in the study. Folate oligos with annealing temperatures and product sizes were indicated.

Gene	Primers sequences (5'-3')	Melting temperature (°C)	PCR fragment size (bp)
External primers			
*folA2-F*	CTACATTTGGAATGTATTGCACT	51	913
*folA2-R*	ATGCTCTATAACATTATGCACTCG	53	
*folC2-F*	TATGATTCCATATTTGGGCTGCG	55	2237
*folC2-R*	CGGKTTACGAATATTATTCGGTCC	54	
*folE2-F*	TACCTCCTAACMTTCGGGCAG	57	1280
*folE2-R*	GATTCCATCCATAAATGTGCSGG	56	
*folKP2-F*	ACCTATCACCCCTTGTGGGTCACA	62	1400
*folKP2-R*	ATCACCGGTTTCAGGAGTGCATGG	61	
*ptpS2-F*	ATCSTCAATTTGAATGCAGGCAG	56	1385
*ptpS2-R*	CGTGTTCGTATGTCACGCTT	56	
Internal primers			
*bioC-F*	TTCTTATTYGGTGAAGCAAT	49	578
*bioC-R*	TATCGRAGTGCYRAWATTAC	48	
*folA-F*	CAGATGCCTTGGTCTTATAC	50	367
*folA-R*	GCCATTCTGCTAAAAGATTA	48	
*folC-F*	RCTCTTTCTGCCTTCCAAAT	53	677
*folC-R*	GAATTATGGCARGTTTGTGA	50	
*folE-F*	ATTCATTGGTGAAGATCCWA	49	409
*folE-R*	ACGCATHGCATACARCTAT	51	
*folKP-F*	TTTCTATARGCAGTAATTTAGGY	49	662
*folKP-R*	ATAATCKGCAATTAATCGTA	45	
*ptpS-F*	TAAACCTCAACAAAACAA	44	416
*ptpS-R*	ATGATMAAATGTACTCGTCGY	51	

Amplification of the five folate genes of *Rickettsia* species phylotype G021 in *I*. *pacificus* was performed using a gradient PCR protocol: initial denaturation at 95°C for 5 minutes, followed by 40 cycles of denaturation at 95°C for 30 seconds, annealing at the temperatures between 50°C and 60°C for 30 seconds, and extension at 72°C for 1 minute and 30 seconds. Each folate gene was amplified by PCR using two sets of primers: external primers and internal primers (Table 1); only external primers amplified the whole open reading frames of each folate gene. A gradient PCR thermocycler, MJ Research PTC-200 (MJ Research, St. Bruno, Canada), was used to establish optimal annealing temperature for amplification of the genes. Each PCR reaction is composed of 2 μL 50 ng/μL *I*. *pacificus* genomic DNA, 1 μL 5 μM forward and reverse primers, 10 μL GoTaq® Green Master Mix (Promega, Madison, WI) and 6 μL PCR grade water. Two positive controls were used in PCR: *Rickettsia montanensis* DNA, provided by Dr. David H. Walker at the University of Texas Medical Branch, and *I*. *scapularis* DNA, extracted from *I*. *scapularis* ticks obtained the National Tick Research and Education Resource at Oklahoma State University. Amplification was confirmed by gel electrophoresis using 1% agarose. DNA bands corresponding to the expected gene sizes were used for cloning.

### Cloning and plasmid DNA purification

Resulting amplicons of the folate genes from PCR were ligated into StrataClone™ PCR Cloning vector *Escherichia coli* Psc-A-amp/kan plasmid (Agilent Technologies, La Jolla, CA). A blue-white screen of the clones containing the insert was performed to detect successful ligation. White colonies on LB agar plates containing ampicillin and X-gal were re-streaked and then grown in LB broth with ampicillin overnight at 37°C. Plasmid DNA was extracted from the cells using the Wizard Plus SV Miniprep kit (Promega, Madison, WI). Eco RI restriction enzyme digest and gel electrophoresis were used to confirm the sizes of the clone inserts.

### Sequencing and sequence analyses

The extracted plasmid DNA was sequenced through Elim Biopharmaceuticals (https://www.elimbio.com/) using M13 primer and sequence-specific primers. Ten clones for each gene were sequenced. The GenBank accession numbers for the folate genes of phylotype G021 are KT225568 for *folA* gene, KT225569 for *folC* gene, KT225570 for *folE* gene, KT225571 for *folKP* gene, and KT225572 for *ptpS* gene. The folate genes of phylotype G021 were used as the query sequences to perform homology searches using BLASTN. Kyoto Encyclopedia of Genes and Genomes (KEGG) was used as a reference knowledge base to interpret the completed *Rickettsia* genomes for the presence of the folate biosynthetic pathway.

Physical gene maps were generated in order to determine where on the genome the folate genes are located and if the folate genes of phylotype G021 and other *Rickettsia* species are organized in an operon. Genes from the species with closest identity to those from phylotype G021 were used to select the organisms to be represented in the gene map. NCBI’s GenBank was used to locate the gene loci within each organism’s complete genome. Arrows were used to represent ORFs. Each gene adjacent to the folate genes (upstream and downstream) was mapped the same way to show the physical clustering of gene loci. The directionality of each gene was based on the orientation displayed on GenBank.

Genes from the organisms other than the genus *Rickettsia* with close identity to the *folA* gene of phylotype G021 were also chosen to be represented on the gene map. Using BLASTX with the exclusion of the *Rickettsiaceae* family, the organisms *Wolbachia* endosymbiont of *Drosophila melanogaster* and *Chlamydophila felis* were chosen as members of two of the genera with a total BLAST score of at least 115 (E value less than 2e^-28^), when compared to *folA* of phylotype G021. *Chlamydia trachomatis* was selected due its total BLAST score of 92.8 (E value of 9e-^20^), when compared to *folA* of phylotype G021, and its previous characterization as a folate synthesizing endosymbiont [42]. *Mycobacterium avium* was also selected due to its known role as a folate synthesizing endosymbiont [43].

### Phylogenetic tree constructions

To study evolutionary relationship of phylotype G021 to validated *Rickettsia* species, the database nucleotide collection (nr/nt) from BLAST was used to find homologies to our folate gene sequences. Sequences of the folate genes from phylotype G021, along with homologous folate gene sequences from closely related bacteria, were aligned with ClustalX version 2.0, edited manually using Geneious 8.0 (http://www.geneious.com/), and subjected to phylogenetic analyses by PAUP* 4.0 that is implemented in Geneious 8.0. Jukes and Cantor model was used to determine evolutionary distance values; the values were then used to construct phylograms by a neighbor-joining method [26]. The maximal parsimony method implemented in Geneious 8.0 was also used to confirm the results of the neighbor-joining method.

## Results

Bioinformatics approaches were used to gain insight into biosynthesis of vitamins and cofactors of *R*. *buchneri* in *I*. *scapularis*. Based on MUMmer alignments against complete genomes of *R*. *bellii*, *R*. *conorii*, and *R*. *felis*, 2,903, 7,496, and 7,203 Trace Archive sequences from the *I*. *scapularis* genome project with a minimal nucleotide identity of 70% were identified to be homologous to the three reference *Rickettsia* genome sequences. Overall, MUMmer identified 8,190 unique sequences that are homologous to *Rickettsia* sequences in the Trace Archive (S1 Table). Among the sequence matches, two vitamin biosynthetic pathways were manually annotated by MetaCyc metabolic reconstructions in the genome of *R*. *buchneri* in *I*. *scapularis*, including the vitamin B7 (biotin) and B9 (folate) biosynthetic pathways.

MUMmer alignments and MetaCyc metabolic reconstructions revealed that all folate gene orthologues required for de novo folate biosynthesis are present in the genome of *R*. *buchneri* in *I*. *scapularis*. The five folate genes are *folA*, coding for dihydrofolate reductase (FolA), *folC*, coding for dihydrofolate synthase (or folylpolyglutamate synthase, FolC), *folE*, coding for GTP-Cyclohydrolase I (FolE), the *folKP* gene encoding a bifunctional protein 2-amino-4-hydroxy-6-hydroxymethyldihydropteridine diphosphokinase (FolK) and dihydropteroate synthase (FolP), and *ptpS*, coding for 6-pyruvoyltetrahydropterin synthase (PtpS-III).

**Fig 1:**
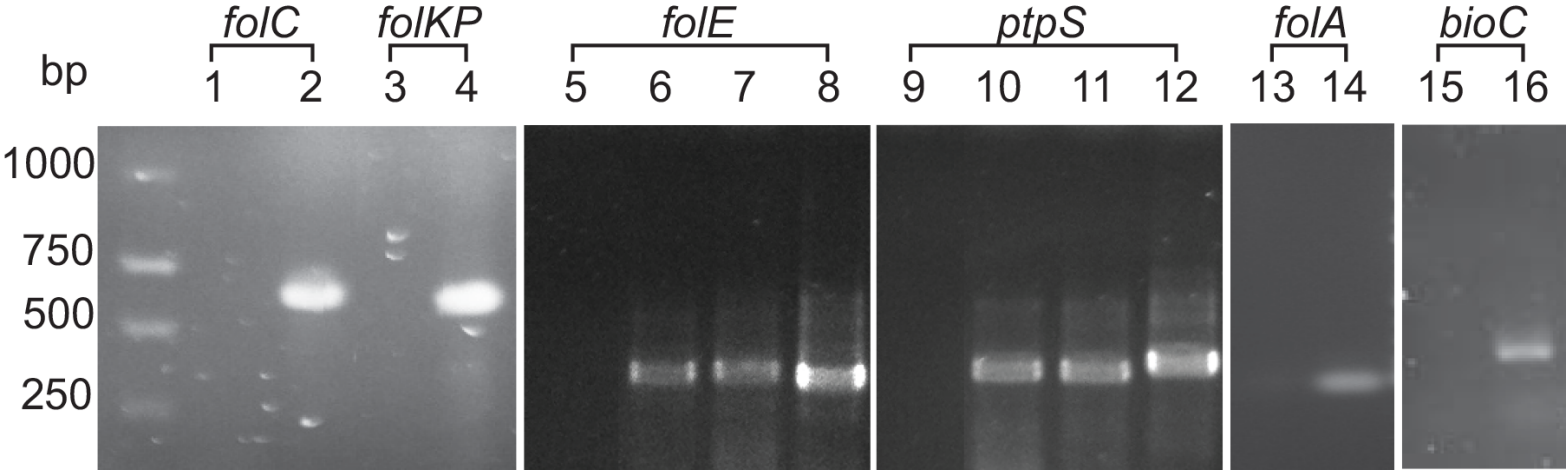
Detection of genes from the folate biosynthetic pathway of *Rickettsia* species phylotype G021 in *Ixodes pacificus* by PCR. *I*. *pacificus* tick genomic DNA was used as the template for PCR amplification of the genes using internal primers (Table 1). The PCR products were electrophoresed and observed by staining with ethidium bromide. Lane 1 and 2: *folC* gene amplification without or with *I*. *pacificus* DNA, respectively; Lane 3 and 4: *folKP* gene amplification without or with *I*. *pacificus* DNA, respectively; Lane 5 and 6: *folE* gene amplification without or with *I*. *pacificus* DNA, respectively; Lane 7 and 8: *folE* gene amplification using *I*. *scapularis* DNA or *Rickettsia montanensis* DNA, respectively; Lane 9 and 10: *ptpS* gene amplification without or with *I*. *pacificus* DNA, respectively; Lane 11 and 12: *ptpS* gene amplification using *I*. *scapularis* DNA and *Rickettsia montanensis* DNA, respectively; Lane 13 and 14: *folA* gene amplification without or with *I*. *pacificus* DNA, respectively. Lane 15 and 16: *bioC* gene amplification without or with *I*. *pacificus* DNA, respectively. bp, base pair.

To test if *Rickettsia* species phylotype G021 has the genetic capacities of biotin and folate biosynthesis, PCR was carried out using degenerate primers designed based on conserved upstream and downstream regions as well as conserved internal sequences of the biotin and folate genes in order to ensure amplifications of complete ORFs of the folate genes of phylotype G021 in *I*. *pacificus* (Table 1). All five genes (*folA*, *folC*, *folE*, *folKP*, and *ptpS*) of the folate biosynthetic pathway were successfully amplified using gene-specific primers. The sizes of the PCR amplicons match the expected sizes of the folate genes (Fig 1). In contrast, only the *bioC* gene (encoding malonyl-CoA O-methyltransferase) in the biotin biosynthetic pathway was amplified by the degenerate primers in this study (Fig 1).

**Fig 2:**
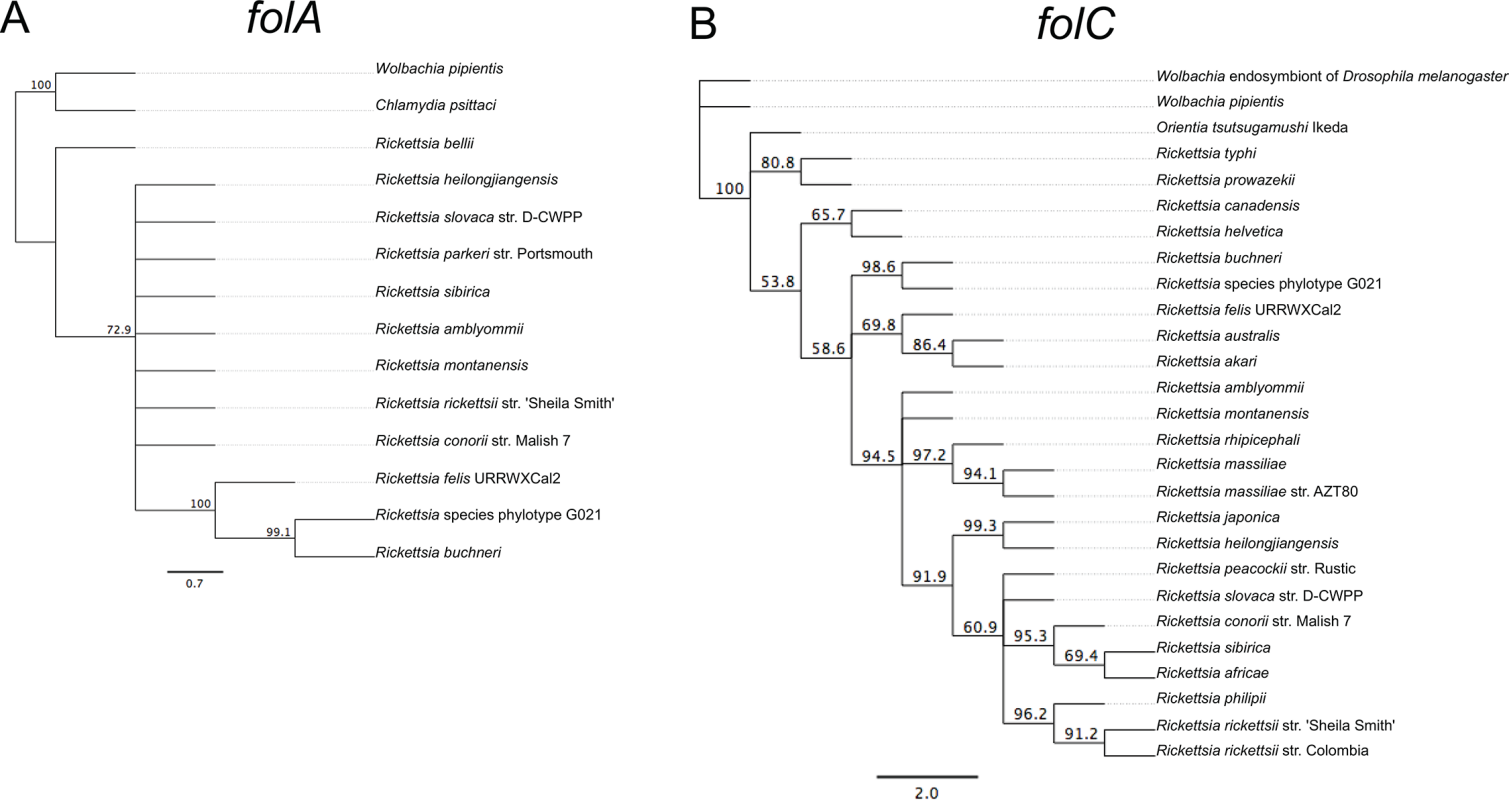
Phylogenetic analyses of the *folA* and *folC* genes of *Rickettsia* species phylotype G021 in *Ixodes pacificus*. (A) Phylogram of the *folA* gene; (B) *folC* gene. The phylogenetic trees were constructed using the Jukes and Cantor model and the neighbor-joining method included in PAUP*4.0 software. Bootstrap values (based on 1,000 replicates) that are greater than 50% are indicated at the nodes.

**Fig 3:**
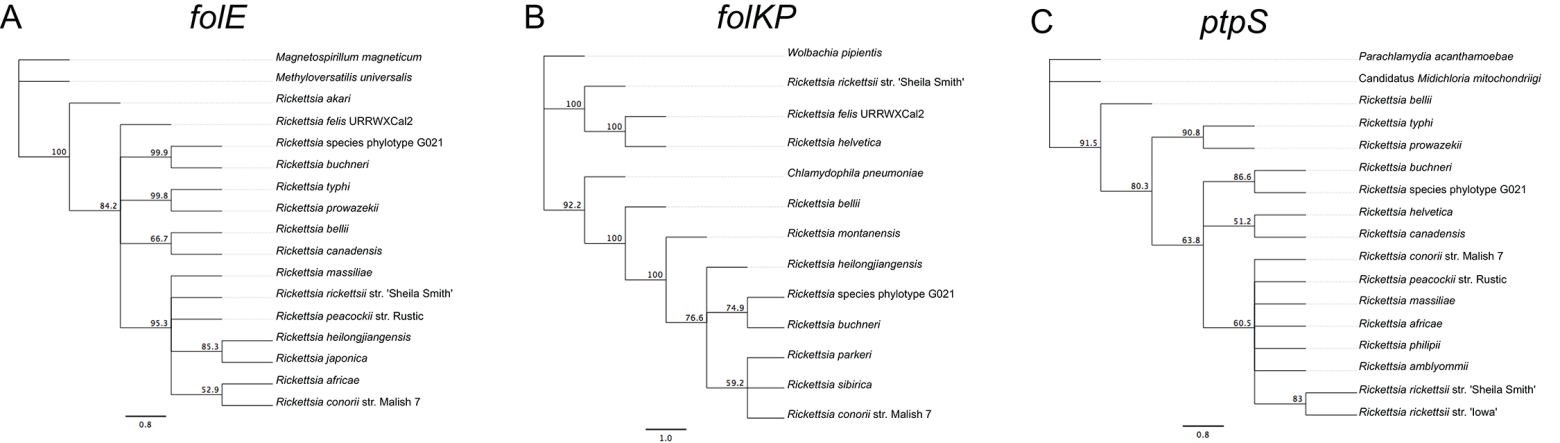
Phylogenetic analyses of the *folE*, *folKP*, and *ptpS* genes of *Rickettsia* species phylotype G021 in *Ixodes pacificus*. (A) Phylogram of the *folE* gene; (B) *folKP* gene; (C) *ptpS* gene. The phylogenetic trees were constructed using the Jukes and Cantor model and the neighbor-joining method included in PAUP*4.0 software. Bootstrap values (based on 1,000 replicates) that are greater than 50% are indicated at the nodes.

Sequencing of ten clones for each folate gene of *Rickettsia* species phylotype G021 showed that the folate genes have bacterial origin and no significant homology with any *Ixodes* genes. BLAST searches revealed that the *folA*, *folC*, *folE*, *folKP*, and *ptpS* genes of phylotype G021 possess 98.6%, 98.8%, 98.9%, 98.5% and 99.0% nucleotide sequence identity, respective to the corresponding genes of *R*. *buchneri* in *I*. *scapularis* [44]. Phylogenetic tree constructions analyses showed that the folate genes of phylotype G021 in *I*. *pacificus* and homologous genes from various *Rickettsia* species from the spotted fever group are monophyletic (Figs 2 and 3). The phylogenetic trees based on the sequences of the *folA* and *folC* genes showed that phylotype G021 in *I*. *pacificus* is clustered with *R*. *buchneri* in *I*. *scapularis* and other spotted fever group rickettsiae as well as *R*. *bellii* for both *folA* and *folC* genes, although the *folC* gene is also clustered with the *folC* gene from the typhus group rickettsiae. However, the *folA* gene from phylotype G021 is on a branch that is distinct from the *folA* gene from other genera of bacteria, such as *Wolbachia* species and *Chlamydophila felis*. Likewise, the *folC* gene from phylotype G021 falls in a clade that is separated from other genera of bacteria, such as *Orientia*, *Neorickettsia*, *Wolbachia*, and others (Fig 2). In the phylogenetic trees based on the *folE*, *folKP*, and *ptpS* nucleotide sequences, phylotype G021 was again in a cluster of *Rickettsia* endosymbionts in *Ixodes* ticks with strong bootstrap support. Phylotype G021 in *I*. *pacificus* and *R*. *buchneri* in *I*. *scapularis* are placed on the same branch with other spotted fever group rickettsiae (Fig 3).

Subsequent bioinformatic analyses based on sequences of the clone DNA confirmed that the folate biosynthetic pathway-coding genes exist in the genome of *Rickettsia* species phylotype G021. Specifically, the *folA*, *folC*, *folE*, *folKP*, and *ptpS* genes from phylotype G021 have 642, 1,281, 573, 1,329, and 417 nucleotide ORFs, respectively, that encode 213, 426, 190, 442, and 138 amino acids, respectively, with predicted protein molecular masses of 24.65, 47.4, 21.81, 50.56, and 16.13 kDa, respectively. The upstream region of each folate gene from phylotype G021 was studied with the aim of identifying cis-acting control elements. We have identified a putative ribosome-binding site (identical or one nucleotide mismatch to the prokaryotic consensus sequences AGGAGG or AGGA of the ribosome-binding site) around 8 nucleotides immediately preceding each folate gene. Using Softberry BPROM, we have identified a putative *E*. *coli σ* ^70^-like promoter that is located from 31 to 51 nucleotides upstream of the translational start codon of *folE* gene. However, no putative *E*. *coli* σ^70^-like promoters were identified within 67, 54, 60, and 77 sequenced nucleotides that are upstream of the translation start site of the *folA*, *folC*, *folKP*, and *ptpS* genes of the sequenced clones, respectively.

**Fig 4:**
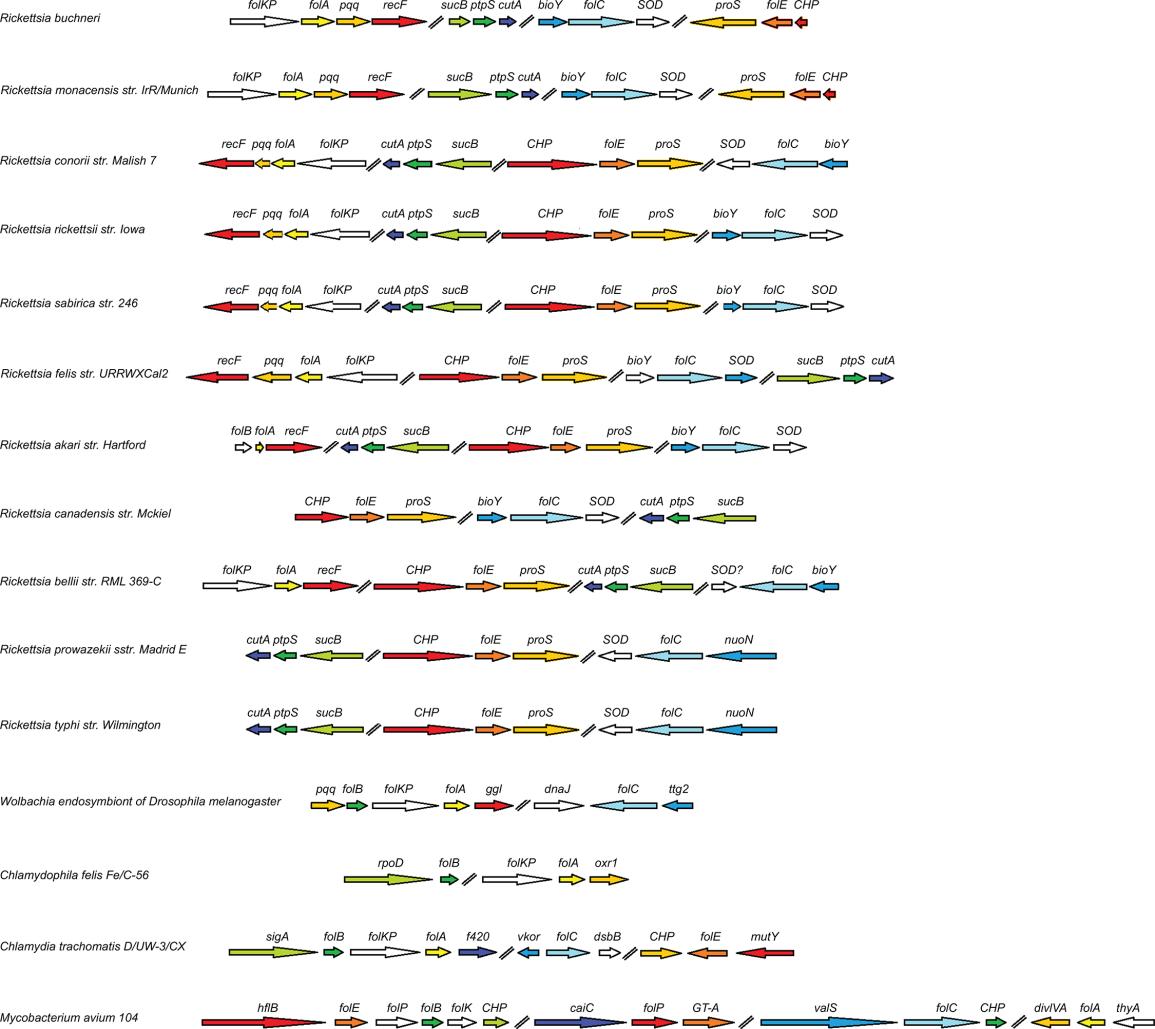
Physical clustering of folate biosynthetic genes across *Rickettsia* genus and related bacterial species. ORFs and coding strand are indicated by arrows. The lengths of the arrows represent the lengths of the genes, with a scale of 0.1 inch = 100 bp. Adjacent arrows represent proximal loci, while the double lines between some arrows represent non-adjacency between loci. The names above the arrows represent the gene name; *CHP* gene encodes for conserved hypothetical protein with no known function; Abbreviations not described in the text: *proS*, prolyl-tRNA synthetase; *sod*, superoxide dismutase; *bioY*, biotin synthesis BioY protein; *cutA*, divalent cation tolerance protein; *sucB*, dihydrolipoamide succinyltransferase; *nuoN*, NADH dehydrogenase subunit N; *ggl*, gamma-glutamate ligase; *dnaJ*, molecular chaperone DnaJ; *ttg2*, toluene tolerance protein; *rpoD*, RNA polymerase sigma factor; *oxr1*, oxidoreductase 1; *thyA*, thymidylate synthetase; *mutY*, adenine DNA glycosylase; *dsbB*, disulfide bond formation protein; *vkor*, vitamin K epoxide; *f420*, coenzyme f420 hydrogenase; *sigA*, RNA polymerase sigma factor RpoD; *hflB*, ATP-dependent metalloprotease; *caiC*, crotonobetaine/carnitine-CoA ligase; *GT-A*, beta-1,4-N-acetyl-galactosaminyl transferase 1; *valS*, valyl-tRNA synthetase; *divlVA*, cell division protein DivlVA.

In contrast to a monocistronic organization of the other folate genes, the *folA* and *folKP* gene loci are polycistronic; the mRNA is made of *folKP-folA-pqq-recF* genes (the *pqq* gene encodes for pyrroloquinoline synthesis protein and *recF* encodes for a DNA replication and repair protein). Although the polycistronic *folKP-folA* gene organization is found in many bacteria [45, 46], BLAST searches showed that the *folKP-folA-pqq-recF* polycistronic organization is only present in the *Rickettsia* genus. *Wolbachia*, a closely related genus, have a different genetic organization of the *folKP-folA* genes, indicating that the two genera employed different evolutionary strategies for acquiring the folate genes (Fig 4) [47].

The KEGG pathway database was used as a reference knowledge base to see if the folate biosynthetic pathway is present in other *Rickettsia* species. Among 44 annotated *Rickettsia* genomes, other than *R*. *buchneri* in *I*. *scapularis* and *Rickettsia* species phylotype G021 in *I*. *pacificus*, fifteen *Rickettsia* species have five folate genes for de novo folate biosynthesis (Fig 4). Specifically, two *Rickettsia* species (*R*. *bellii RML 369* and *R*. *bellii BSU8S-389)* in the *Rickettsia belli* group and thirteen *Rickettsia* species (*R*. *conorii* subsp. Israelensis, *R*. *felis* str. LSU-Lb, *R*. *rickettsii Shelia Smith*, *R*. *rickettsii Arizona*, *R*. *rickettsia Colombia*, *R*. *rickettsii Hauke*, *R*. *rickettsii Brazil*, *R*. *rickettsia Hino*, *R*. *rickettsii Morgan*, *R*. *rickettsii R*, *R*. *heilongjiangensis*, *R*. *montanensis*, and *R*. *parkeri*) in the spotted fever group have all five folate genes that are required for de novo folate biosynthesis. In contrast, genome sequences from other *Rickettsia* species revealed that the *folKP* gene is lost and only some of the five genes of the folate biosynthetic pathway are present. Specifically, some *Rickettsia* species possess *folC*, *folE*, and *ptpS* genes only, whereas some other species possess only *folA*, *folC*, *folE*, and *ptpS* genes. The genetic capacity for de novo folate biosynthesis is not related to bacterial pathogenicity since both pathogens (such as *R*. *rickettsii*) and endosymbionts (such as *Rickettsia* species phylotype G021 and *R*. *buchneri*) have all five folate genes. Interestingly, all typhus group rickettsiae sequenced so far (two *R*. *typhi* genotypes, ten *R*. *prowazekii* genotypes) have *folC*, *folE*, and *ptpS* genes, but *folA*, *folK*, and *folP* genes are not present in their genomes (Fig 4).

## Discussion

Our findings that *R*. *buchneri* in *I*. *scapularis* has all five genes required for the synthesis of folate were based on MUMmer alignments and MetaCyc metabolic reconstructions using the NCBI Trace Archive data from the *I*. *scapularis* genome sequencing project [34, 35]. The bioinformatics analyses were carried out before the complete annotation of the genome of *I*. *scapularis* was released in 2012 (https://www.vectorbase.org/news/ixodes-scapularis-gene-set-iscaw12-released). Our study demonstrated that *Rickettsia* species phylotype G021 in *I*. *pacificus* has the genetic capacity to de novo synthesize tetrahydrofolate. In contrast, only the *folA*, *folC*, and *folE* genes were correctly annotated by the *I*. *scapularis* genome sequencing project (http://www.ncbi.nlm.nih.gov/genome/1908). So far, the genome of *R*. *buchneri* ISO7^T^ strain [48] has been sequenced but has not been annotated completely (http://www.ncbi.nlm.nih.gov/genome/38153).

Tetrahydrofolate and other folate derivatives are the most essential components for cell growth [49]. Folate is vitamin B9 and is needed by all eukaryotic and prokaryotic organisms for the one-carbon metabolism in nucleotide synthesis and in the methylation of DNA, RNA, proteins, and phospholipids [50–52]. Folate insufficiency causes megaloblastic anemia and neural tube defects, such as spina bifida, in humans [53, 54]. Larvae of *Drosophila melanogaster* showed prolonged development when fed with a diet containing antibiotics and near-zero folate [47]. Deletions of the genes of the folate biosynthetic pathway in *E*. *coli* lead to the production of non-viable phenotypes [55, 56]. Animals cannot synthesize folate, and must therefore obtain it either through diet or mutualistic associations with microbes [16, 47]. The tick genome project revealed that ticks lack genetic capacity for de novo folate synthesis [34, 35]. Interestingly, ticks have the ability to survive as long as a year until the next life stage. Since ticks feed exclusively on nutrient poor (especially vitamin B9 and vitamin B7) vertebrate blood [16], they must obtain folate through non-dietary means.

**Fig 5:**
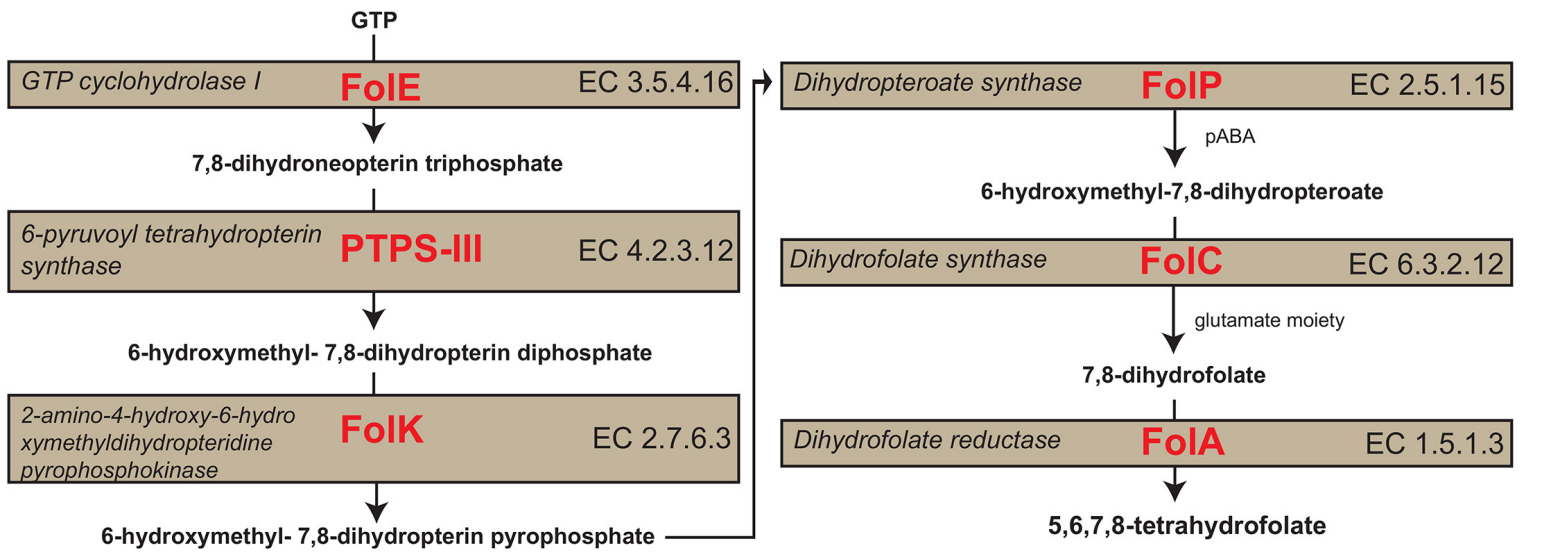
The proposed pathway of folate biosynthesis by *Rickettsia* species phylotype G021 in *Ixodes pacificus*. The diagram shows the sequence of intermediates produced from the enzymatic reactions that constitute the pathway for synthesizing folate. The precursor, GTP, is transformed and combined with pABA and glutamate, which eventually leads to the reduced cofactor tetrahydrofolate.

We propose a model for de novo folate biosynthesis by phylotype G021. Guanosine-5'-triphosphate (GTP), from tick hosts or bacterial sources, is catalyzed by a series of enzymatic actions of the FolA, FolC, FolE, FolKP, and PtpS-III proteins from phylotype G021 to synthesize tetrahydrofolate (Fig 5). First, the FolE protein (GTP-Cyclohydrolase I) synthesizes 7,8-dihydroneopterin triphosphate, a pterin-ring molecule, from GTP [57–59]. Then the PTPS-III protein (6-pyruvoyltetrahydropterin synthase) cleaves the side chain of 7,8-dihydroneopterin triphosphate to form 6-hydroxymethyl-7,8-dihydropterin diphosphate [60, 61]. Then the FolKP bifunctional enzyme (2-amino-4-hydroxy-6-hydroxymethyldihydropteridine diphosphokinase and dihydropteroate synthase) joins the pterin ring to para-aminobenzoic acid (pABA) to synthesize 6-hydroxymethyl-7,8-dihydropteroate [62]. A glutamate moiety is then added to 6-hydroxymethyl-7,8-dihydropteroate by FolC (dihydrofolate synthase) to synthesize 7,8-dihydrofolate [63], which is then utilized by the FolA protein (dihydrofolate reductase) to synthesize tetrahydrofolate [43].

Many bacteria and plants as well as lower eukaryotes have a genetic capacity for de novo folate biosynthesis that requires the presence of a *folB* gene. The *folB* gene encodes dihydroneopterin aldolase, an enzyme that catalyzes the substrate 2-amino-4-hydroxy-6-(D-erythro-1,2,3-trihydroxypropyl)-7,8-dihydropteridine to 2-amino-4-hydroxy-6-hydroxymethyl-7,8-dihydropteridine and glycolaldehyde [60, 61]. However, our MUMmer alignments didn’t identify a *folB* orthologue in the genome of *R*. *buchneri* of *I*. *scapularis*. Likewise, the *folB* gene is not present in genomes of many bacteria, including the phyla *Acidobacteria*, *Chloroflexi*, *Firmicutes*, *Planctomycetes*, and *Spirochaetes* [61]. Although non-orthologous enzymes could carry out the function of dihydroneopterin aldolase, or a *folB* gene with a low nucleotide identity (less than the minimum alignment identity of 70% of MUMmer) could be present in the genome, we identified a PtpS orthologue from the Trace Archive sequences of *R*. *buchneri* of *I*. *scapularis* and amplified the *ptpS* gene from phylotype G021. Since both FolB and PtpS-III proteins produce the product 6-hydroxymethyl-7,8 dihydropterin, which is required as a substrate for the FolKP protein, PtpS-III protein is considered as a functional alternative of the FolB protein in the folate biosynthesis pathway of many bacteria and *Plasmodium* species [60] as well as phylotype G021.

Due to the essential functions of folate in cell growth and cell division [50–52], it is not surprising that both pathogenic and endosymbiotic rickettsiae can carry out de novo folate biosynthesis. Having this genetic capacity is important since folate is absolutely required during the period of rapid cell growth and cell division inside hosts, including the engorgement stage after blood meals in arthropod hosts. A readily available source of folate synthesized by phylotype G021 promotes rapid cell proliferation not only for the bacterium but also for *I*. *pacificus* tick. This nutritional interaction between endosymbiotic phylotype G021 and *I*. *pacificus* may explain the predominant presence of the bacterium in *I*. *pacificus* [27, 28]. It should also be noted at this time that many other bacteria belonging to the tick microbiome could also provide folate in addition to phylotype G021. However, the interaction between *I*. *pacificus* and phylotype G021 is an important first step in the study of the folate biosynthesis and provides a framework for identifying other potential bacterial folate producers in the tick microbiome.

Although many *Rickettsia* species from the spotted fever group and *Rickettsia belli* group have the genetic capacity to synthesize folate, none of the twelve sequenced *Rickettsia* species or genotypes from the typhus group possess the *folA*, *folK*, and *folP* genes in their genomes (Fig 4). The missing genes indicate that those *Rickettsia* species lack the genetic capacity for de novo biosynthesis of folate. The major invertebrate hosts for the typhus group *Rickettsia* are lice and fleas [64]. In contract, hard ticks are the main hosts for the spotted fever and *Rickettsia belli* group of *Rickettsia*. It could be true that lice and fleas have other enzymes from either arthropods or other microbial symbionts to convert 6-hydroxymethyl-7,8-dihydropterin to tetrahydrofolate.

Little is known about the nutritional basis for the symbiotic relationship of endosymbionts in ticks, though newly developed systems are beginning to shed light on novel interactions. Kurtti et. al, recently isolated and characterized *Rickettsia buchneri* from tick embryonic cell lines IRE11 and ISE6 [48]. This approach provides a basis to further testing symbiotic interactions of other *Rickettsia* species. Employing a similar methodology, the culturing of *Rickettsia* species phylotype G021 using a tick embryonic cell line could be feasible. Understanding the nutritional interactions between *Rickettsia* species in ticks may help us to eliminate Lyme disease and other tick-borne diseases by suppressing tick population.

## Supporting Information

S1 File SequenceSearch script code. (JAVA)

S2 File NucleotideReader script code. (JAVA)

S1 Table Endosymbiotic rickettsial genes and their encoding proteins identified by the MUMmer alignment software and BLASTX in the Trace Archive of the *Ixodes scapularis* genome sequencing project. Column 1 and 2 are call numbers. Column 3 is the GenBank accession numbers of homologous proteins. Column 4 is the names of the homologous proteins. Column 5 is the names of the bacterial species. Column 6 and 7 are the percentage identity to top BLASTX results. Column 8 is the AT% for the DNA. Column 9 is the sequences obtained by the MUMmer alignment.
(XLS)
